# Whole-Genome Methylation Analysis Revealed ART-Specific DNA Methylation Pattern of Neuro- and Immune-System Pathways in Chinese Human Neonates

**DOI:** 10.3389/fgene.2021.696840

**Published:** 2021-09-13

**Authors:** Zongzhi Liu, Wei Chen, Zilong Zhang, Junyun Wang, Yi-Kun Yang, Luo Hai, Yuan Wei, Jie Qiao, Yingli Sun

**Affiliations:** ^1^Central Laboratory, National Cancer Center/National Clinical Research Center for Cancer/Cancer Hospital & Shenzhen Hospital, Chinese Academic of Medical Sciences and Peking Union Medical College, Shenzhen, China; ^2^Department of Thoracic Surgery, National Cancer Center/National Clinical Research Center for Cancer/Cancer Hospital & Shenzhen Hospital, Chinese Academy of Medical Sciences and Peking Union Medical College, Shenzhen, China; ^3^University of Chinese Academy of Sciences, Beijing, China; ^4^Center for Reproductive Medicine, Department of Obstetrics and Gynecology, Peking University Third Hospital, Beijing, China; ^5^Key Laboratory of Assisted Reproduction, Ministry of Education, Beijing, China; ^6^Beijing Key Laboratory of Reproductive Endocrinology and Assisted Reproduction, Beijing, China; ^7^Peking-Tsinghua Center for Life Sciences, Peking University, Beijing, China; ^8^CAS Key Laboratory of Genome Sciences and Information, Beijing Institute of Genomics, Chinese Academy of Sciences/China National Center for Bioinformation, Beijing, China; ^9^Tianjin Novogene Bioinformatic Technology Co., Ltd.,, Tianjin, China

**Keywords:** human offspring, differentially methylated regions, housekeeping gene, assisted reproductive technology, DNA methylation pattern, imprinted gene

## Abstract

The DNA methylation of human offspring can change due to the use of assisted reproductive technology (ART). In order to find the differentially methylated regions (DMRs) in ART newborns, cord blood maternal cell contamination and parent DNA methylation background, which will add noise to the real difference, must be removed. We analyzed newborns’ heel blood from six families to identify the DMRs between ART and natural pregnancy newborns, and the genetic model of methylation was explored, meanwhile we analyzed 32 samples of umbilical cord blood of infants born with ART and those of normal pregnancy to confirm which differences are consistent with cord blood data. The DNA methylation level was lower in ART-assisted offspring at the whole genome-wide level. Differentially methylated sites, DMRs, and cord blood differentially expressed genes were enriched in the important pathways of the immune system and nervous system, the genetic patterns of DNA methylation could be changed in the ART group. A total of three imprinted genes and 28 housekeeping genes which were involved in the nervous and immune systems were significant different between the two groups, six of them were detected both in heel blood and cord blood. We concluded that there is an ART-specific DNA methylation pattern involved in neuro- and immune-system pathways of human ART neonates, providing an epigenetic basis for the potential long-term health risks in ART-conceived neonates.

## Introduction

Assisted reproductive technology (ART) involves fertilizing a human egg *in vitro* and the transplantation of the resulting embryo into the uterus for conception (Van Voorhis, 2007; Belva et al., 2016; Boulet et al., 2016; De Geyter et al., 2018). Decades after the first successful application of ART in humans, over 8 million infants have been born with ART assistance worldwide (Wade et al., 2015; Rao et al., 2018; Fauser, 2019; Leaver and Wells, 2019). ART has become well-accepted and popular in recent years (Liu et al., 2015; Simopoulou et al., 2018; Gleicher et al., 2019). Epidemiological and animal experiments show that the early stage of fetal development is particularly sensitive to changes in the environment and that the environmental abnormalities suffered during this period may lead to problems later in life (Faulk and Dolinoy, 2011; Hanson and Gluckman, 2014; Grandjean et al., 2015; Heindel et al., 2017; Li T. et al., 2019). During this sensitive period, adverse environmental stimulation may affect cell proliferation and lineage differentiation by affecting normal epigenetic reprogramming processes, leading to abnormal epigenetic modification levels and permanent changes in gene expression patterns (Yamazaki et al., 2003; Hanson and Gluckman, 2014; Nelissen et al., 2014; Koot et al., 2016). ART treatments, such as exposure to culture medium and gamete or embryo freezing, may affect DNA methylation reprogramming and embryonic development (de Waal et al., 2015; Canovas et al., 2017b; Mani and Mainigi, 2018; Argyraki et al., 2019). Zoological and embryological studies have revealed that ART can affect the DNA methylation pattern and the expression of imprinted genes in mouse, pig, and bovine embryos (Wright et al., 2011; de Waal et al., 2014; Anckaert and Fair, 2015; Salilew-Wondim et al., 2015; Canovas et al., 2017a; Hattori et al., 2019). Additionally, epidemiological studies have reported the abnormal development of the immune system (Tan et al., 2016; Kollmann et al., 2017; Pathare et al., 2017), increased risk of neurological diseases, the presence of metabolic abnormalities, and the presence of congenital anomalies in ART-assisted human offspring, including autism spectrum disorders, intellectual disability, specific congenital heart defects, cardiovascular disease, and metabolic disorder (Sandin et al., 2013; Tararbit et al., 2013; Guo et al., 2017; Liu et al., 2017).

More and more observations of rising health risks in ART-conceived neonates have linked ART technology to potential epigenetic abnormities (Tararbit et al., 2013; Guo et al., 2017). Previous works only focused on the epigenetic influence of ART on a limited numbers of genes. Recent works reported the impact of ART on genome-wide DNA methylation. Nevertheless, the DNA methylation of ART processes has not been fully elucidated, and parental background has not been considered. Several studies showed that ART was associated with diverse DNA methylation changes in human offspring (Melamed et al., 2015; Castillo-Fernandez et al., 2017; DeAngelis et al., 2018; Menezo et al., 2019). However, these observations were primary and these studies analyzed DNA methylation from cord blood data, which contained maternal cell contamination (Lo et al., 1996, 2000) affecting the results of the methylation data analyses (Houseman et al., 2012; Bakulski et al., 2016; Jones et al., 2017; Lin et al., 2018). Therefore, a mixture of cord blood samples may result in high background and unclear data of DNA methylation.

**FIGURE 1:**
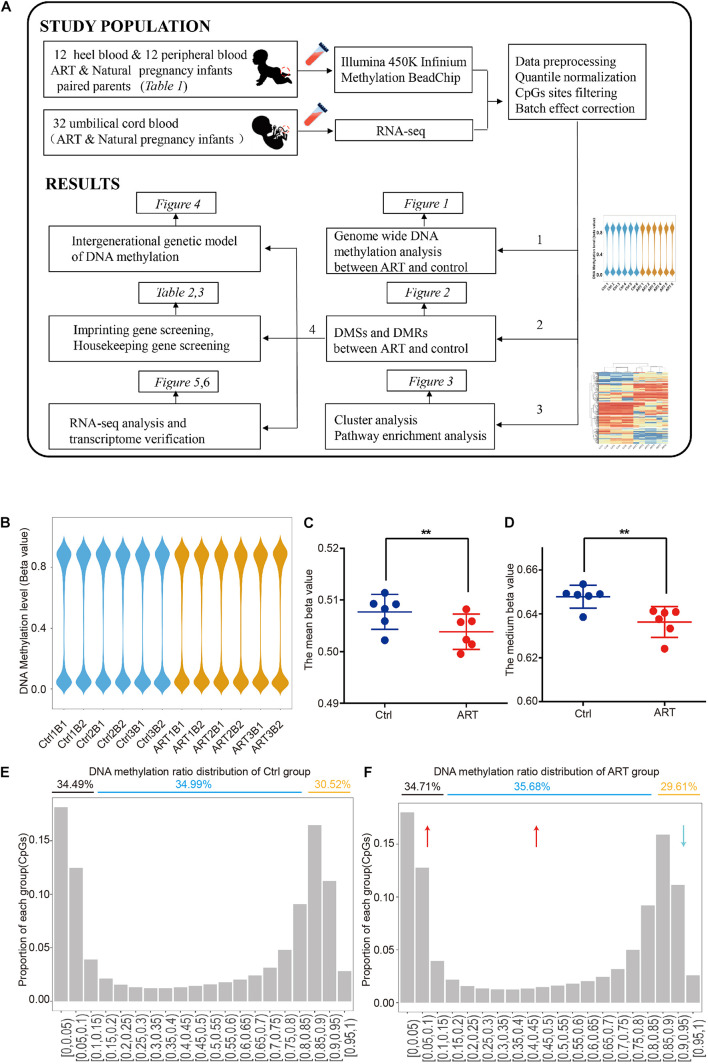
Genome-wide DNA hypomethylation pattern in assisted reproductive technology (ART)-conceived infants. **(A)** Graphical overview of the study design. **(B)** The distribution of DNA methylation showed that the two groups had a similar pattern at the genome level. Mean **(C)** and median **(D)** DNA methylation levels. DNA methylation ratio distribution of the control **(E)** and ART group **(F)**.

Herein, as shown in Figure 1A, by using the heel blood of the ART-conceived newborns and removing the background DNA methylation level of parents, we are able to perform a more accurate and reliable analysis of DNA methylation with low background noise. Heel prick blood sampling enables us to identify ART-specific DNA methylation pattern changes precisely, objectively, and accurately.

## Materials and Methods

### Editorial Policies and Ethical Considerations

All samples were obtained in the Center for Reproductive Medicine, Peking University Third Hospital. All blood samples were obtained after written informed patient consent and were fully anonymized. The studies involving human participants were reviewed and approved by The Reproductive Study Ethics Committee of Peking University Third Hospital (approved protocol no. 201752-044). The participants’ parents and legal guardians provided written informed consent to participate in this study.

### Participants and Study Design

We collected information on three families who had assisted reproductive pregnancies and three families who normal pregnancies. All families had twins by cesarean birth. The heel blood of neonates was collected 3 days after birth. The peripheral blood of parents was stored in an EDTA blood collection vessel. The Illumina Methylation 450K array was performed on all samples. Meanwhile, we analyzed the RNA-seq data we recently uploaded to the GEO database which we collected from umbilical cord blood of 32 newborns who underwent IVF-ET, IVF-FET, ICSI-ET, ICSI-FET, and normal pregnancy.

### Characteristics of the ART and Control Groups

The gestational age of the newborns at birth was 34–38 weeks. All the newborns in the three ART families and three control families were delivered by cesarean section. Neither parent had a history of familial hereditary diseases, these details are shown in Table 1.

**TABLE 1:** Detailed information on Control and assisted reproductive technology (ART) samples.

	Control 1	Control 2	Control 3	Control 4	Control 5	Control 6	ART1	ART2	ART3	ART4	ART5	ART6
Family ID	Control Family 1	Control Family 1	Control Family 2	Control Family 2	Control Family 3	Control Family 3	ART Family 1	ART Family 1	ART Family 2	ART Family 2	ART Family 3	ART Family 3
Cesarean birth	Yes	Yes	Yes	Yes	Yes	Yes	Yes	Yes	Yes	Yes	Yes	Yes
Gestational age	36	36	37	37	34	34	37	37	38	38	34	34
Infant body length	47 cm	47 cm	48 cm	48 cm	43 cm	41 cm	47 cm	45 cm	49 cm	48 cm	44 cm	44 cm
Infant weight	2560 g	2600 g	2600 g	2810 g	2250 g	1810 g	2740 g	2220 g	2940 g	2830 g	1980 g	2010 g
Sex	Female	Female	Male	Male	Male	Male	Female	Female	Female	Female	Male	Male
Father’s age	–	–	–	–	26	26	40	40	33	33	–	–
Mother’s age	28	28	30	30	25	25	38	38	32	32	27	27
Mother’s weight	82 kg	82 kg	66.5 kg	66.5 kg	82 kg	82 kg	68 kg	68 kg	74.5 kg	74.5 kg	77 kg	77 kg
Mother’s height	161 cm	161 cm	163 cm	163 cm	172 cm	172 cm	167 cm	167 cm	169 cm	169 cm	162 cm	162 cm
Weight gain during pregnancy	18.5 kg	18.5 kg	18.5 kg	18.5 kg	20 kg	20 kg	7.5 kg	7.5 kg	15 kg	15 kg	18 kg	18 kg
Hereditary diseases	None	None	None	None	None	None	None	None	None	None	None	None

### DNA Extraction

Genomic DNA was extracted using the QIAamp DNA Mini Kit (QIAGEN 51304, Germany) according to the manufacturer’s instructions.

### Methylation Microarray Analysis

Genomic DNA (1 μg) was bisulfite-converted using the EZ DNA Methylation-Gold Kit (ZYMO RESEARCH, D5005, FosterCity, California, United States). Then DNA was whole-genome-amplified, enzymatically fragmented, purified, and applied to the Illumina Infinium Methylation 450K array according to the Illumina methylation protocol.

### Analysis of the Methylation Microarray Data

DNA methylation files were processed and normalized by R software packages using the “Illumina Methylation Analyzer (IMA)” package. For each of the samples, CpG sites with a detection *p*-value less than 0.05 were excluded from the analysis. In addition, probes with SNPs or their single base extension, X chromosome and Y chromosome at the CpG site were excluded. Differences in global DNA methylation levels between the ART and control groups were analyzed by the Wilcoxon test. The standard DMSs between the ART and control groups were δ beta| > 0.2 and *p* < 0.05 (Wilcoxon test). DMRs analysis used QDMR Tutorial software (Zhang et al., 2011).

Chromosome distribution differences were analyzed by the Wilcoxon test. Cluster analysis and visualization were performed using pheatmap (R package). Heel blood DNA methylation pathway enrichment analysis was performed by IPA. Information on imprinted genes was obtained from the gene imprint website.^1^ Information on housekeeping genes was obtained from Eisenberg and Levanon (2013).

### Analysis of RNA-Seq Data

Downloaded raw fastq data were firstly processed using Trimmomatic (version 0.36) to remove the library adapter, low-quality bases, and reads smaller than 50 bases. The retained reads were mapped to the Homo sapiens reference genome (human GRCh38/hg38) using STAR (version 2.5.3a) with the default parameters, and read counting was performed using featureCounts (version 1.6.3). Finally, DEseq2 (version 1.26.0) was used to obtain the normalized count matrix for all samples. GO enrichment analysis was carried out using the R/Bioconductor package ChIPseeker (Houseman et al., 2012; version 1.10.3).

## Results

### The Whole Genome-Wide Methylation Differences in ART-Conceived and Naturally Conceived Neonates

In order to compare the differences of global methylation level between ART and naturally conceived newborns, we first analyzed the genome-wide methylation patterns. The overall DNA methylation beta value showed similar distribution patterns among all samples (Figure 1B and Supplementary Figure 1). However, the global DNA methylation level was lower in the ART group ($\bar{\beta }$ART = 0.504 ± 0.003 versus $\bar{\beta }$ctrl = 0.508 ± 0.003). Further analysis of the mean (Figure 1C) and median (Figure 1D) DNA methylation beta values of individuals confirmed this observation.

After the methylation beta level (0–1) was divided into approximately 20 intervals, the results revealed that such hypomethylation in the ART group was mainly caused by the decreased ratio in CpG sites with high DNA methylation levels (methylation beta level > 0.85), with 29.61% in the ART group and 30.52% in the control group, and the increased ratio in the CpG site with low and medium DNA methylation levels (methylation beta level < 0.85), with 70.39% in the ART group and 69.48% in the control group (Figures 1E,F).

### Differential Methylation Sites Were Enriched in Important Pathways of the Immune and Nervous Systems

**FIGURE 2:**
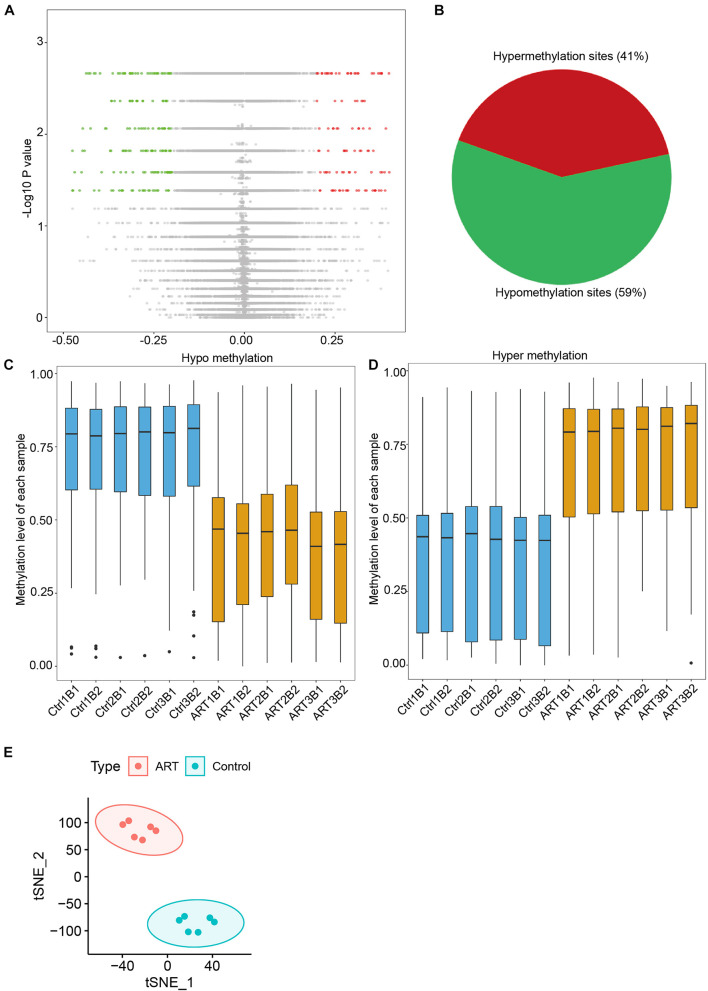
Differential methylation analysis showed that the number of hypomethylation sites in ART-conceived infants was significantly higher than the number of hypermethylation sites. **(A)** Distribution of differences in DNA methylation levels between the two groups of infants. **(B)** The ratio of sites with hypomethylation differences and hypermethylation differences was 59:41. Distribution of methylation levels at different sites of hypomethylation **(C)** and hypermethylation **(D)**. **(E)** tSNE analysis of differentially methylated sites (DMSs).

As shown in Figure 2A, a total of 301 differential methylation sites (DMSs) were screened as described in the “Materials and Methods” part. The number of hypomethylated DMSs counted for 178, and the number of hypermethylated DMSs was 123 (59 versus 41%; Figure 2B), which was consistent with the analysis of demethylation at the genome-wide level mentioned. The beta value analysis of the DMSs for each sample showed this tendency more clearly. The mean beta values of differential hypomethylation sites in the ART group were 0.418 ± 0.032 and 0.730 ± 0.007 in the control group (Figure 2C). The mean beta values of differential hypermethylation sites in the ART group were 0.702 ± 0.018 and 0.372 ± 0.0124 in the control group (Figure 2D). Through t-SNE analysis, the DMSs can significantly divide the ART and control infants into two groups (Figure 2E).

**FIGURE 3:**
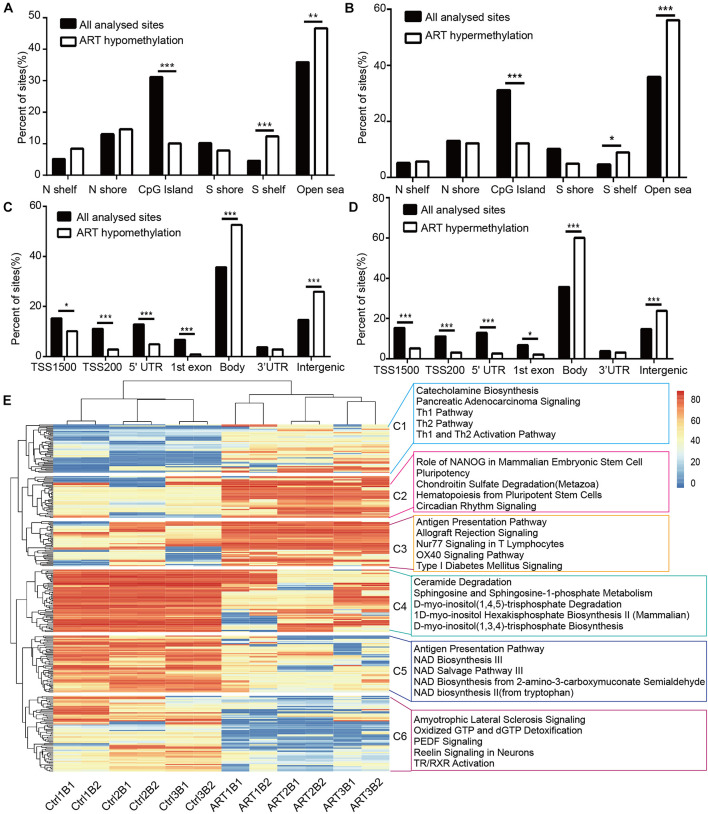
Non-randomized effects of ART on DNA methylation patterns were associated with early infant development. The distribution of hypomethylated **(A)** and hypermethylated DMSs **(B)** affected by ART in relation to the nearest CpG island. The distribution of hypomethylated **(C)** and hypermethylated DMSs **(D)** affected by ART in relation to the nearest genes. **(E)** Heatmap derived from supervised cluster analysis of DMSs. The red color in the heatmap indicates hypermethylation values, and blue indicates hypomethylation values.

DMSs were significantly enriched in the S shelf and open sea based on their relationship with CpG islands (Figures 3A,B) and gene bodies and intergenic regions based on the nearest genes (Figures 3C,D). Specifically, they were represented in the CpG islands, TSS1500, TSS200, 5′UTR, and 1st exon. These findings further indicated that the ART-assisted and control groups shared similar methylation patterns with specific differences.

Furthermore, the DMSs we identified could be used to classify the ART group and control group by supervised cluster analysis. Six ART infants were clustered together, and six control infants were grouped together at the top of the cluster tree (Figure 3E). Further, we discovered several consistent patterns when DMSs were clustered into six subgroups (C1–C6) based on supervised clusters. The methylation levels of DMSs in the C1, C4, and C5 subgroups were relatively conservative in all six control infants (C1: hypomethylation in nearly all sites, C4 and C5: hypermethylation in nearly all sites) but with high randomness and diversity in the six ART infants (different samples had different methylation levels at the same site). In contrast, the methylation levels of DMSs in the C2, C3, and C6 subgroups were greatly heterogeneous among the six control infants (different samples had different methylation levels at the same site) but with high consistency in the six ART infants (C2 and C3: hypermethylation in nearly all sites, C6: hypomethylation in nearly all sites). Additionally, all loci were statistically analyzed for the degree of dispersion to confirm our results. In parts C1, C4, and C5, the scattering degree (SD) value of the ART group was larger, while that of the control group was more conservative. In parts C2, C3, and C6, the SD value of the ART group was smaller, while that of the control group was larger (Supplementary Table 1). Furthermore, ingenuity pathway analysis (IPA) showed that DMSs were enriched in key pathways, e.g., (1) the “Ceramide Degradation” and “Catecholamine Biosynthesis” pathways were well associated with nervous system development and nerve signals transfer; (2) the “Antigen Presentation Pathway,” “Th1 Pathway,” and “Th2 Pathway” were important pathways related to immune system establishment; (3) “NAD Biosynthesis III,” “NAD Salvage Pathway III,” and “NAD Biosynthesis” played key roles in metabolic development. Importantly, the pathway analysis results were consistent with clinical manifestations, such as with a cognitive development problem and a higher risk of autism in ART-conceived infants (Fountain et al., 2015; Rumbold et al., 2017; Berntsen et al., 2019).

### Immune- and Nervous-System Pathways Were Identified by Differential Methylation Regions Analysis and Cord Blood RNA-Seq

DNA methylation usually functioned in a region, we identified differentially methylated regions (DMRs) to find their functional association. Interestingly, DMRs analysis showed similar results with DMSs. The obtained DMRs were highly enriched in the regulation of neuron differentiation processes, antigen presentation, and other important developmental pathways (Supplementary Figure 2). Moreover, 10 of the 301 DMSs with the most remarkable differences in methylation beta values could be used to divide the two groups in the principal component analysis (PCA) (Supplementary Figure 3). These 10 most susceptible sites included 7 genes, namely, *ZNF137*, *TAP2*, *RBM28*, *NUDT1*, *NMNAT3*, *EIF3E*, and *AFAP1* (Supplementary Table 2). Among the above genes, four genes were involved in neurological and immune-related functions: (1) *TAP2* was related to the expression of major histocompatibility complex (MHC) class I molecules and the development of insulin-dependent diabetes mellitus (Qu et al., 2007; Qiu et al., 2015; Praest et al., 2018); (2) *RBM28* was related to progressive neurological defects and endocrinopathy (Nousbeck et al., 2008); (3) NUDT1 was related to neurodegeneration (Pudelko et al., 2017; Haruyama et al., 2019); (4) *NMNAT3* encoded a member of the nicotinamide/nicotinic acid mononucleotide adenylyltransferase family and played a neuroprotective role as a molecular chaperone (Galindo et al., 2017). Collectively, these results suggested that DNA methylation in ART offspring could be changed and that these changes were enriched in development-related pathways, particularly in the nervous, immune, and metabolic systems.

### The Opposite Genetic Pattern of DNA Methylation Between ART and Normal Pregnancy Infants

**FIGURE 4:**
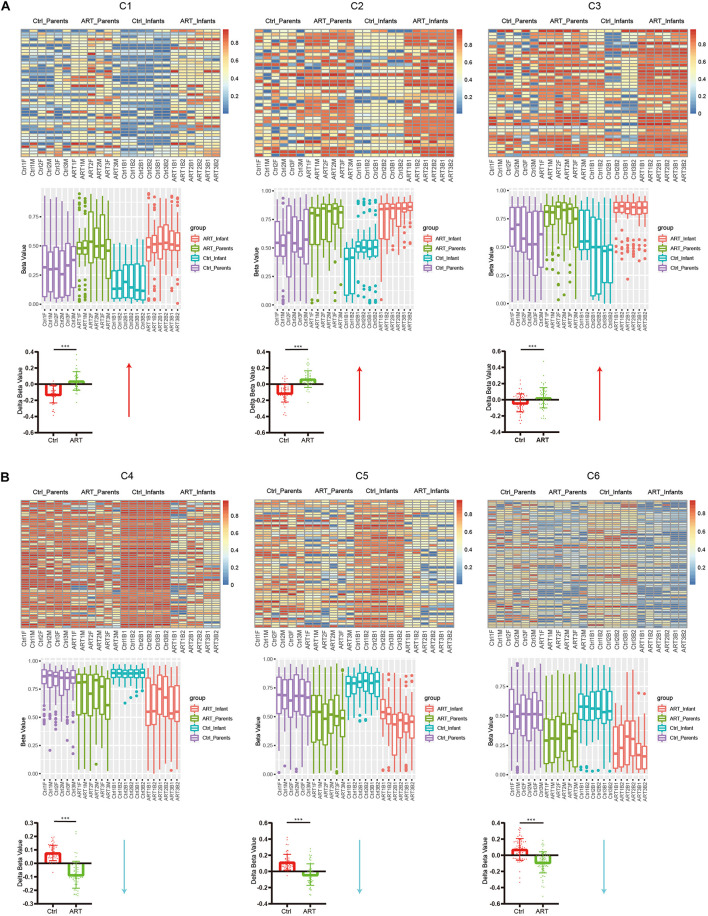
The opposite genetic pattern of DNA methylation between ART and normal pregnancy infants. **(A)** In C1–C3, the methylation level of normal pregnancy infants tended toward hypomethylation compared with their parents, however, there was a trend of hypermethylation in ART infants compared with their parents. **(B)** In C4–C6, the methylation level of normal pregnancy infants tended toward hypermethylation compared with their parents, however, there was a trend of hypomethylation in ART infants compared with their parents. All the opposite genetic patterns had significant statistical difference (*p* < 0.001).

We have confirmed that the DNA methylation pattern of ART infants was different from that of normal pregnant infants through DMSs, DMRs, and DEGs analyses, further, we want to confirm whether these epigenetic differences are influenced by parental heredity. As shown in Figure 4, we analyzed C1–C6 separately and combined them with their parents, then we found that the difference of DNA methylation pattern between ART and natural pregnancy infants also existed in their parents (heatmap, C1–C6), however, the genetic pattern of ART infants was opposite compared to normal pregnancy infants (boxplot, C1–C6 and histogram C1–C6). When we removed the background DNA methylation level of parents, we found that in C1–C3 (Figure 4A) the methylation level of normal pregnancy infants tended toward hypomethylation compared with their parents, however, there was a trend of hypermethylation in ART infants compared with their parents. In C4–C6 (Figure 4B), the methylation level of normal pregnancy infants tended toward hypermethylation compared with their parents, however, there was a trend of hypomethylation in ART infants compared with their parents. All the opposite genetic patterns had significant statistical difference (*p* < 0.001).

### The Discovery of Imprinted Genes and Housekeeping Genes Changes Can Be Found in RNA-Seq of Cord Blood

As described above, ART could specifically affect the conservativeness of the DNA methylation pattern. Imprinted genes and housekeeping genes were conserved in genetic processes and played important roles in development. In a comparison involving the imprinted and housekeeping gene database, we identified 3 DMSs located in 2 imprinted genes (Table 2) and 28 DMSs located in 26 housekeeping genes (Table 3). The two imprinted genes included: (1) *NTM*, which encoded a protein of the IgLON family with specific expression in the brain that promotes neurite outgrowth and adhesion via a homophilic mechanism (Li et al., 2015; Maruani et al., 2015); (2) *BRUNOL4*, which was related to variable splicing of precursor RNA and its editing and normal function of the nervous system (Wang et al., 2016; An et al., 2019). Among the housekeeping genes, *CASP7*, *RBM28*, and *FEZ2* were associated with the maintenance of nerve function (Nousbeck et al., 2008; Choudhury et al., 2015; Hapairai et al., 2017). CMPK1 and INPP5A were related to metabolic function (Zhu et al., 2018; Li G. et al., 2019). *GALNS* and *TAPBP* were involved in the innate immune system and MHC class I-mediated antigen processing and presentation (Williams et al., 2000, 2002; Park et al., 2004; Tamarozzi et al., 2014).

**TABLE 2:** Analysis of imprinted genes affected in ART.

Target ID	CHR	MAPINFO	Gene name	Diff (ART-N)	*P*-value
cg09663736	11	131554122	*NTM*	−0.20	0.026
cg13077366	18	34908626	*BRUNOL4*	−0.22	0.004
cg20094343	18	34917603	*–*	−0.27	0.015

**TABLE 3:** Analysis of housekeeping genes affected in ART.

Target ID	CHR	MAPINFO	Gene name	Diff (ART-N)	*P*-value
cg12501287	10	134411480	*INPP5A*	0.20	0.015
cg16645815	10	134556992	*INPP5A*	–0.25	0.004
cg16542356	7	1121190	*C7orf50*	–0.20	0.026
cg23496178	7	1142861	*C7orf50*	–0.26	0.041
cg00023507	6	33276465	*TAPBP*	–0.33	0.002
cg00999163	1	47799638	*CMPK1*	0.26	0.041
cg01128042	10	115465924	*CASP7*	–0.47	0.041
cg02379549	6	36887307	*C6orf89*	0.31	0.026
cg03119308	7	127950724	*RBM28*	0.68	0.002
cg05385718	2	242693323	*D2HGDH*	0.28	0.015
cg05398700	14	102677141	*WDR20*	–0.23	0.015
cg06314883	6	5404958	*FARS2*	0.21	0.041
cg08136432	16	88902276	*GALNS*	0.38	0.026
cg08603678	8	109235928	*EIF3E*	0.69	0.002
cg08912652	11	130779479	*SNX19*	0.31	0.041
cg12604331	1	156906485	*ARHGEF11*	0.29	0.002
cg13143872	2	200778865	*C2orf69*	–0.31	0.002
cg14060113	19	18054643	*CCDC124*	–0.34	0.041
cg14497649	4	528497	*PIGG*	–0.30	0.002
cg14609104	10	111989324	*MXI1*	0.41	0.009
cg1542132	5	153372524	*FAM114A2*	–0.53	0.002
cg16241932	6	157876915	*ZDHHC14*	0.35	0.026
cg17004290	4	108853384	*CYP2U1*	–0.45	0.015
cg24976563	14	24587638	*DCAF11*	0.36	0.041
cg25282454	1	1158325	*SDF4*	0.31	0.002
cg25465065	1	156198365	*PMF1*	–0.36	0.026
cg26303777	1	230311676	*GALNT2*	–0.24	0.026
cg06634576	2	36782386	*FEZ2*	–0.32	0.015

**FIGURE 5:**
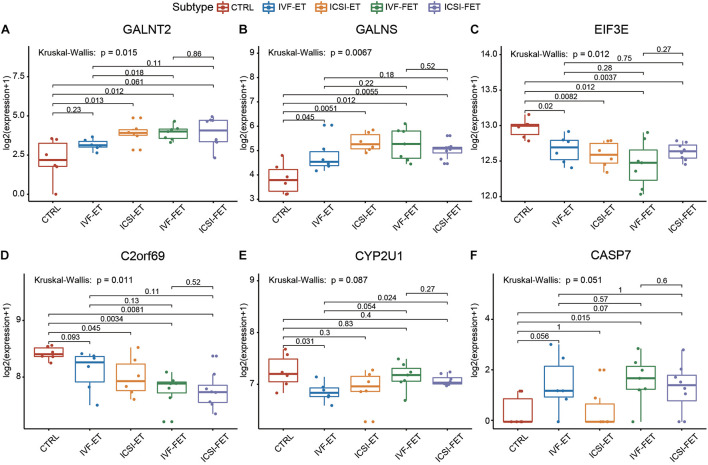
The housekeeping genes and imprinting genes in DMSs was confirmed by RNA-seq. The expression profiles of imprinted genes and housekeeping genes in the four ART subgroups: IVF-ET, IVF-FET, ICSI-ET, and ICSI-FET, six genes had significant differences in all ART subgroups.

**FIGURE 6:**
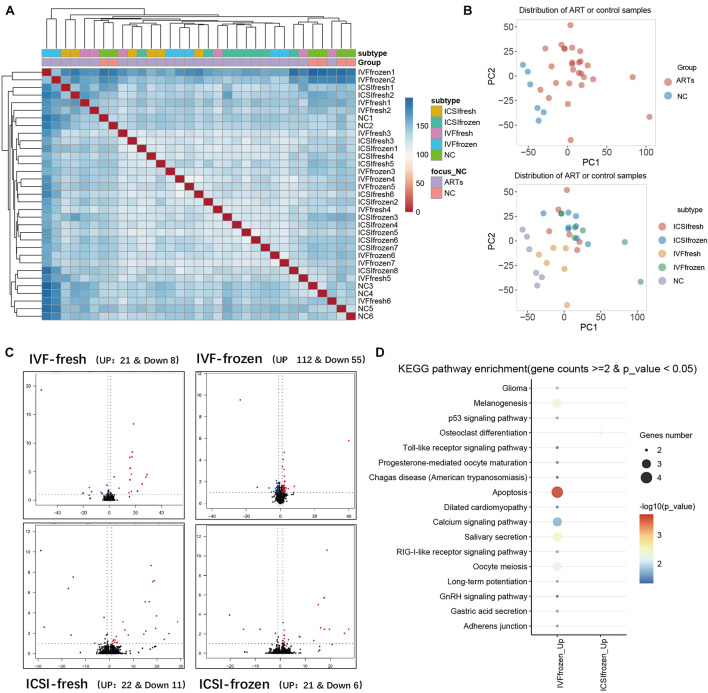
Analysis of RNA-seq in umbilical cord blood of different types of ART and natural pregnancy infants. **(A)** Hierarchical clustering analysis of RNA seq data. **(B)** Principal component analysis (PCA) analysis of ART and natural pregnancy infants. **(C)** Differentially expressed genes (DEGs) analysis of IVF-fresh and IVF-frozen. **(D)** KEGG pathway analysis of IVF-frozen and ICSI-frozen.

To further verify the above imprinted and housekeeping gene, we used our recently published RNA-sequencing data (GSE136849) to analyze the gene expression. The ART-conceived pregnancy families were further divided into four subgroups based on the type of ART applied, including the *in vitro* fertilization-embryo transfer (IVF-ET), *in vitro* fertilization and frozen-thawed embryo transfer (IVF-FET), intracytoplasmic sperm injection-embryo transfer (ICSI-ET), and intracytoplasmic sperm injection and frozen-thawed embryo transfer (ICSI-FET) subgroups. Each ART subgroup had its technical details, but they all intervened on embryonic cells *in vitro*. As shown in Figure 5 and Supplementary Figure 4, the gene expression pattern of the above four ART subgroups was different from that of the control group. Among them, six genes were significantly different from the control group in all ART subgroups, namely *GALNT2*, *GALNS*, *EIF3E*, *C2ORF69*, *CYP2U1*, and *CASP7*, respectively. Among the above genes, *GALNT2* was involved in glucose and lipid metabolism; *EIF3E* was highly associated with the survival of human glioblastoma cells; *CYP2U1* transcripts were most abundant in the thymus and the brain, indicating a specific physiological role for *CYP2U1* in these tissues. Next, we did hierarchical cluster analysis on the transcriptome (Figure 6A), PCA results showed that that the expression profiles of the control group and ART newborns could be significantly divided into two groups (Figure 6B), the number of DEGs between the IVF frozen and natural pregnancy groups was relatively large (Figure 6C). They were highly enriched in pathways involving autophagy and sialic acid secretion (Figure 6D). At the same time, we found that the immune and nervous system-related pathways also had significant statistical differences, which justifies the results of our heel blood methylation pathway analysis (Supplementary Figure 5).

## Discussion

ART treatment can affect the epigenomes of offspring, such as aberrant DNA methylation (Tan et al., 2016; Castillo-Fernandez et al., 2017; El Hajj et al., 2017; Ghosh et al., 2017), most of the studies addressing these changes use the cord blood, which has an unwanted maternal background. Moreover, most of these studies are not whole genome-wide. To demonstrate the specific effect caused by ART, we used heel blood to compare the DNA methylation patterns of ART and naturally conceived newborns.

Our main findings were as follows: (1) ART can affect the conservation of DNA methylation in specific genomic regions, and the DMSs are mainly enriched in the function of the nervous system and immune system; (2) our discovery of DMSs can be confirmed by the difference of DMRs, RNA expression level of imprinted genes, and housekeeping genes in ART babies.

Our study reveals that ART infants show a similar global DNA methylation pattern with naturally conceived infants, which is consistent with the results of NirMelamed’s study (Melamed et al., 2015). However, by using cord blood, which adds background noise from the maternal cells, the above work fails to show the specific methylation difference in ART-assisted babies. We screened 301 DMSs in the heel blood of ART-assisted samples. Although the number of loci screened out was relatively small, it actually reflects the real difference in methylation levels between the ART and control groups. It can be explained by the fact that our sample contains only the methylation information of newborns with no environmental influence, and we set up a strict standard for screening. Previous works do not reveal the genes annotated from all 301 DMSs (Supplementary Figure 6; Melamed et al., 2015; Castillo-Fernandez et al., 2017; El Hajj et al., 2017). The differential genes we found were consistent with the gene found by Juan et al., and there have been very few common discoveries among previous works. Furthermore, our data demonstrate that ART shows a specific effect on the reprogramming of DNA methylation patterns in human offspring.

The pathway analysis of DMSs and DMRs shows that the altered DNA methylation patterns are mainly enriched in key pathways in early-stage developmental pathways, such as neurotransmitter secretion, immune system establishment, and NAD metabolism. Quite a few previous works of clinical studies suggest an increased risk of autism spectrum disorders, intellectual disability, specific congenital heart defects, cardiovascular disease, and metabolic disorder in late-stage ART-assisted infants (Sandin et al., 2013; Guo et al., 2017; Liu et al., 2017). It is reasonable to hypothesize that abnormal DNA methylation in ART-assisted offspring is rooted in the effect of DNA methylation reprogramming in the early developmental stage, as suggested by the Developmental Origins of Health and Disease (DOHaD) theory (Berntsen et al., 2019).

Although we used the blood sample analysis differences of DNA methylation between ART infants and natural pregnancy infants, it is reasonable that ART may have an impact on the development of nervous and immune systems. Heel blood contains a large number of white blood cells, it is easier to understand why the results of heel blood would be enriched in the immune system. Meanwhile, analysis pathway in blood and predating the dysregulation of brain tissue is comprehensively used in many different fields. The results of W Esther show that blood can serve as a surrogate marker for the brain. There is a large correlation between blood DNA methylation and brain diseases, a proportion that, although small, was significantly greater than prediction by chance. A subset of peripheral data may represent the methylation status of brain tissue. The results of Chuang et al. (2017) also showed that DNA methylation levels in human blood and saliva is associated with Parkinson’s disease (Walton et al., 2016; Edgar et al., 2017; Zhang et al., 2017). Although we are not yet able to know the mental and nervous system development of these children in the future, our pathway research shows that it is consistent with current known clinical research phenomena.

We also identify three significant DMSs-located imprinted genes and 28 significant DMSs-located housekeeping genes, and these are key genes related to the development and echo the results of pathway enrichment analysis. These results collectively suggest that the ART process potentially influenced the development of the nervous system in progenies, not only by the co-effect of multiple genes of the nerve gene signaling pathway but also by influencing the methylation status of imprinted genes and housekeeping genes. These six genes, *GALNT2*, *GALNS*, *EIF3E*, *C2ORF69*, *CYP2U1*, and *CASP7*, may be the key genes affected by ART technology, the significant differences can be seen in heel blood and umbilical cord blood at the same time in all kinds of ART technology (IVF-ET, IVF-FET, ICSI-ET, and ICSI-FET), meanwhile, they are related to the nervous system and immune system.

Our findings are highly consistent with previous clinical epidemiology data and highlight the epigenetic impact of ART on the nervous system and the immune system during development. Since the first DNA methylation reprogramming starts from the two-cell stage, it may be influenced by the *in vitro* environment. Further investigation to compare the multi-cell stage embryo may help to uncover the underlying mechanism. Due to the great heterogeneity among populations (such as living habits, genetic background, reproductive age, and health status of the parents), a larger cohort is needed to systematically assess and confirm the above-mentioned epigenetic risk in ART-assisted children. At the same time, our research also has some areas that can be further optimized. For example, we can further control the homozygote and heterozygote of the sampling to ensure that our data are more accurate and reliable and expand our sample size to provide better evidence for our conclusion. Also, since the heel blood was harvested 3 days post-natal, there could be some differential methylation marks added to the baby epigenetic marks. At the same time, we can ensure the quality of blood collection as much as possible, so that we can carry out multi-omics analysis of DNA methylation and transcriptome of the same infant to verify the conclusion of our heel blood data and explore the effect of ART technology on DNA methylation and the genetic pattern of newborns more accurately.

## Conclusion

In summary, we found DMRs between ART-assisted and naturally conceived human offspring at the whole genome-wide level. These DNA methylation variations were enriched in important pathways of the immune system and nervous system.

## Data Availability Statement

The datasets presented in this study can be found in online repositories. The names of the repository/repositories and accession number(s) can be found below: https://www.ncbi.nlm.nih.gov/, GSE142554 https://www.ncbi.nlm.nih.gov/, GSE136849.

## Author Contributions

ZL participated in sample collection, data analysis, and manuscript writing. WC participated in sample collection and manuscript writing. YW participated in sample collection. ZZ, LH, and Y-KY participated in data analysis. JW, JQ, and YS designed the experiment and revised the article. All authors read and approved the final manuscript.

## Conflict of Interest

ZZ was employed by the company Tianjin Novogene Bioinformatic Technology Co., Ltd., China. The remaining authors declare that the research was conducted in the absence of any commercial or financial relationships that could be construed as a potential conflict of interest.

## Publisher’s Note

All claims expressed in this article are solely those of the authors and do not necessarily represent those of their affiliated organizations, or those of the publisher, the editors and the reviewers. Any product that may be evaluated in this article, or claim that may be made by its manufacturer, is not guaranteed or endorsed by the publisher.

## Footnotes

1
http://www.geneimprint.com/site/gene

## Supplementary Material

Supplementary Figure 1 Correlation analysis of DNA methylation in the heel blood of the two groups of newborns. The *X*-axis represents the methylation value of each site in the ART group, and the *Y*-axis represents the methylation value of each point in the control group. Two groups have a significant correlationship *r*^2^ = 0.9973796, *p* = 2.2e-16.

Supplementary Figure 2 Differentially methylated regions (DMRs) pathway analysis. DMRs pathway analysis showed the main different pathways, which were enriched in key pathways of immune-system and neuro-system, such as the Antigen Presentation Pathway, OX40 Signaling Pathway, Neuroprotective Role of THOP1 in Alzheimer’s Disease et al.

Supplementary Figure 3 Assisted reproductive technology (ART)-conceived and naturally conceived infants could be divided into two groups by the ten most susceptible DNA methylation sites. The two components could be divided into two groups by the principal component analysis: yellow represents the ART group; Blue represents the control group.

Supplementary Figure 4 House keeping genes and imprinting genes in DMSs was confirmed by RNA-seq. The expression profiles of *GALNT2*, *GALNS*, *EIF3E*, *C2ORF69*, *CYP2U1*, and *CASP7* in the four ART subgroups: IVF-ET, IVF-FET, ICSI-ET, and ICSI-FET, these six genes had significant differences in all ART subgroups.

Supplementary Figure 5 Differentially expressed genes (DEGs) pathway analysis of different types of ART and natural pregnancy infants. DEGs pathway analysis showed the main different pathways, which were enriched in key pathways of immune-system and neuro-system, such as regulation of T cell activation, regulation of neural precursor cell proliferation and neuroblast proliferation et al.

Supplementary Figure 6 Venn diagram showing the relationship with the reported genes. Venn diagram showed there were few overlaps in the differently methylated genes that have been reported.
