# Supplementary figures and images for: Whole-Genome Methylation Analysis Revealed ART-Specific DNA Methylation Pattern of Neuro- and Immune-System Pathways in Chinese Human Neonates
Source: Front Genet. 2021 Sep 13;12:696840. doi: 10.3389/fgene.2021.696840 (PMC8473827; doi:10.3389/fgene.2021.696840)

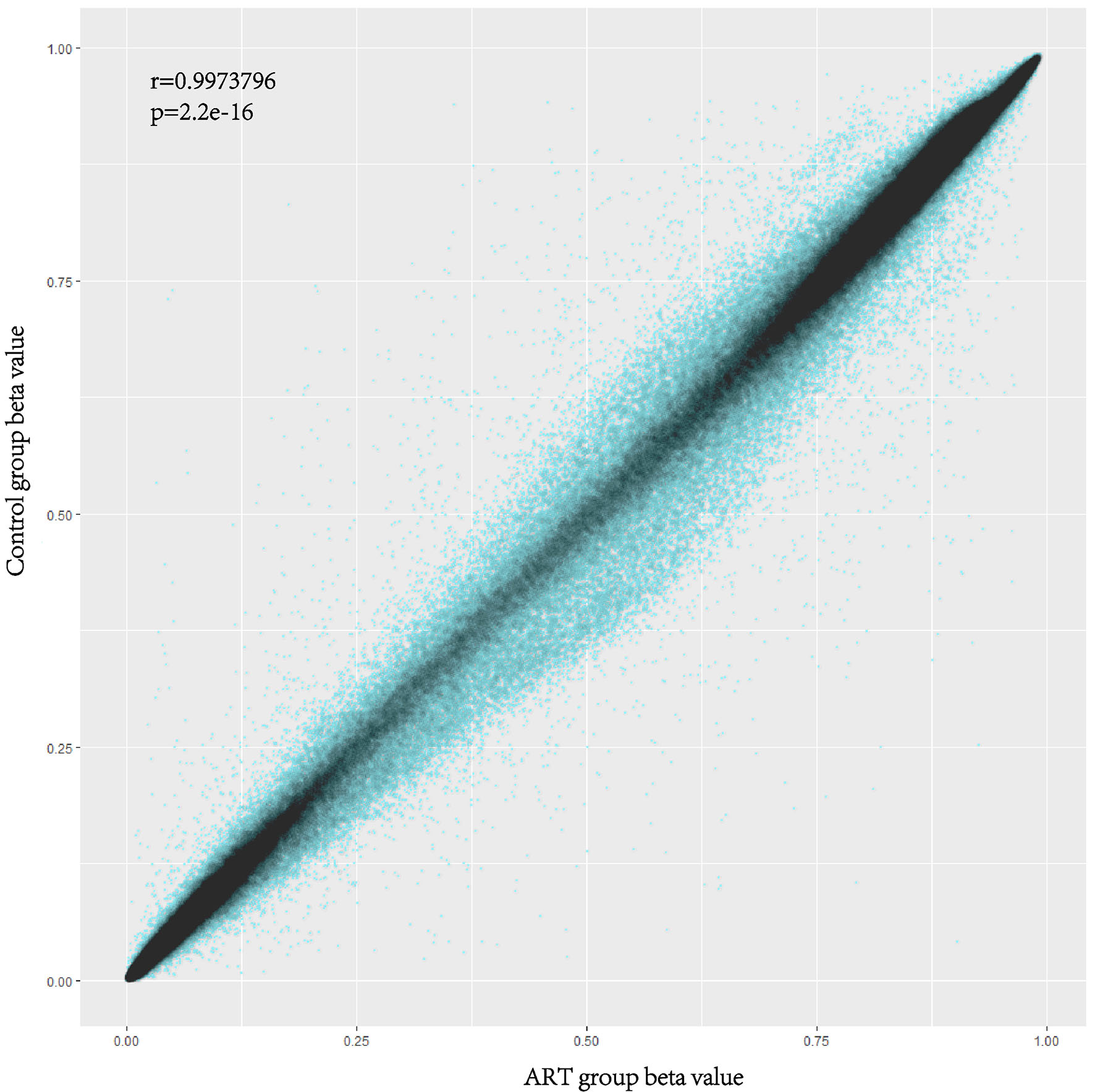

Supplement: Supplementary Figure 1 — Correlation analysis of DNA methylation in the heel blood of the two groups of newborns. The X-axis represents the methylation value of each site in the ART group, and the Y-axis represents the methylation value of each point in the control group. Two groups have a significant correlationship r2 = 0.9973796, p = 2.2e-16. [file Image_1.JPEG]

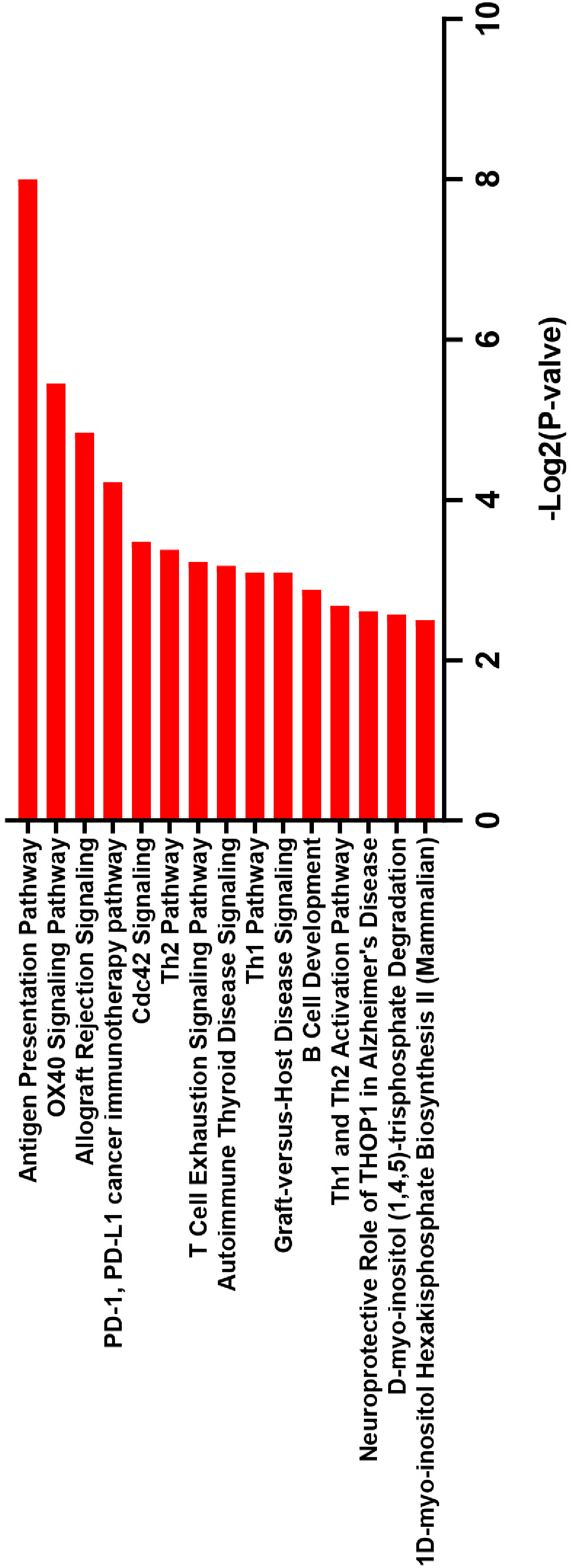

Supplement: Supplementary Figure 2 — Differentially methylated regions (DMRs) pathway analysis. DMRs pathway analysis showed the main different pathways, which were enriched in key pathways of immune-system and neuro-system, such as the Antigen Presentation Pathway, OX40 Signaling Pathway, Neuroprotective Role of THOP1 in Alzheimer’s Disease et al. [file Image_2.JPEG]

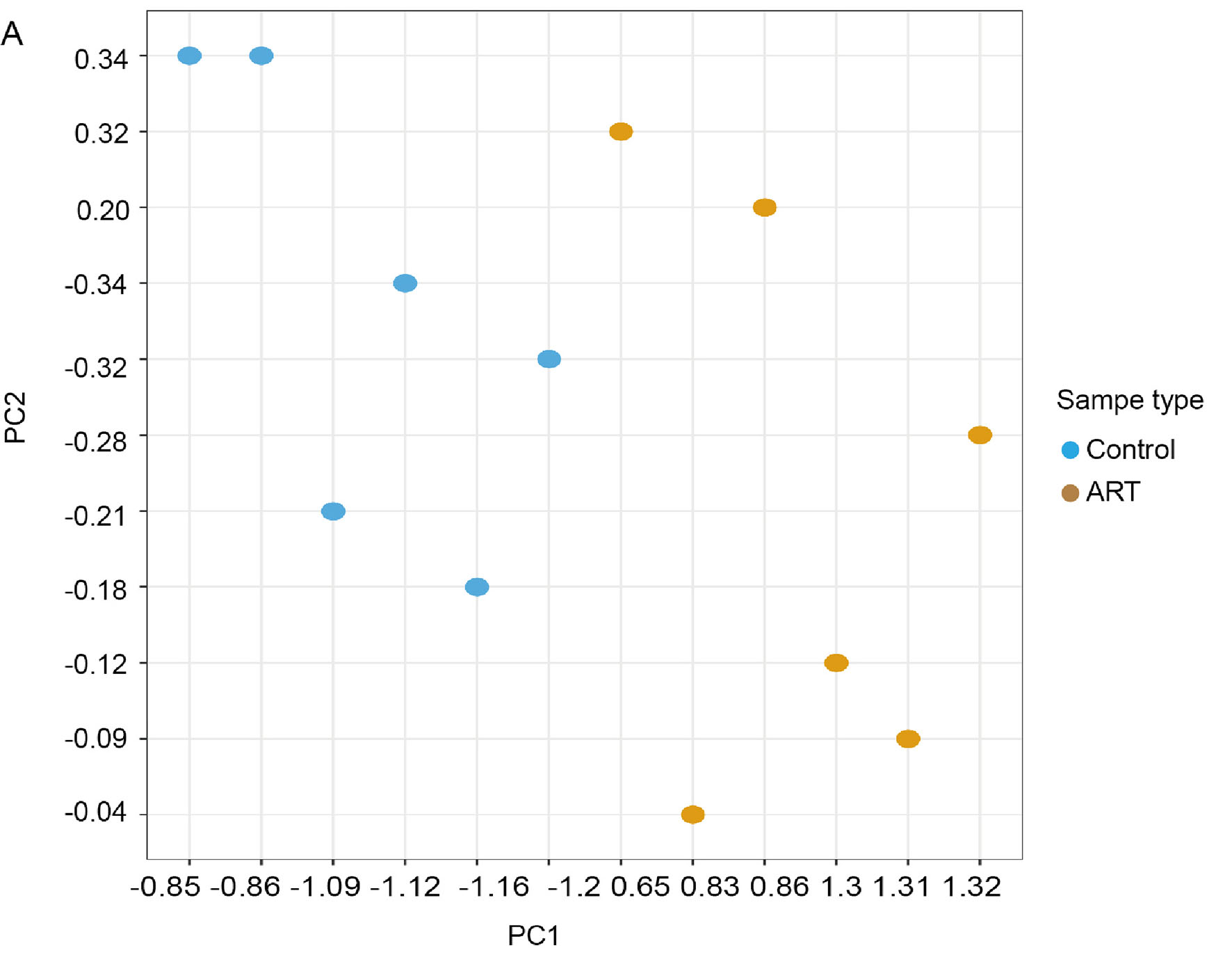

Supplement: Supplementary Figure 3 — Assisted reproductive technology (ART)-conceived and naturally conceived infants could be divided into two groups by the ten most susceptible DNA methylation sites. The two components could be divided into two groups by the principal component analysis: yellow represents the ART group; Blue represents the control group. [file Image_3.JPEG]

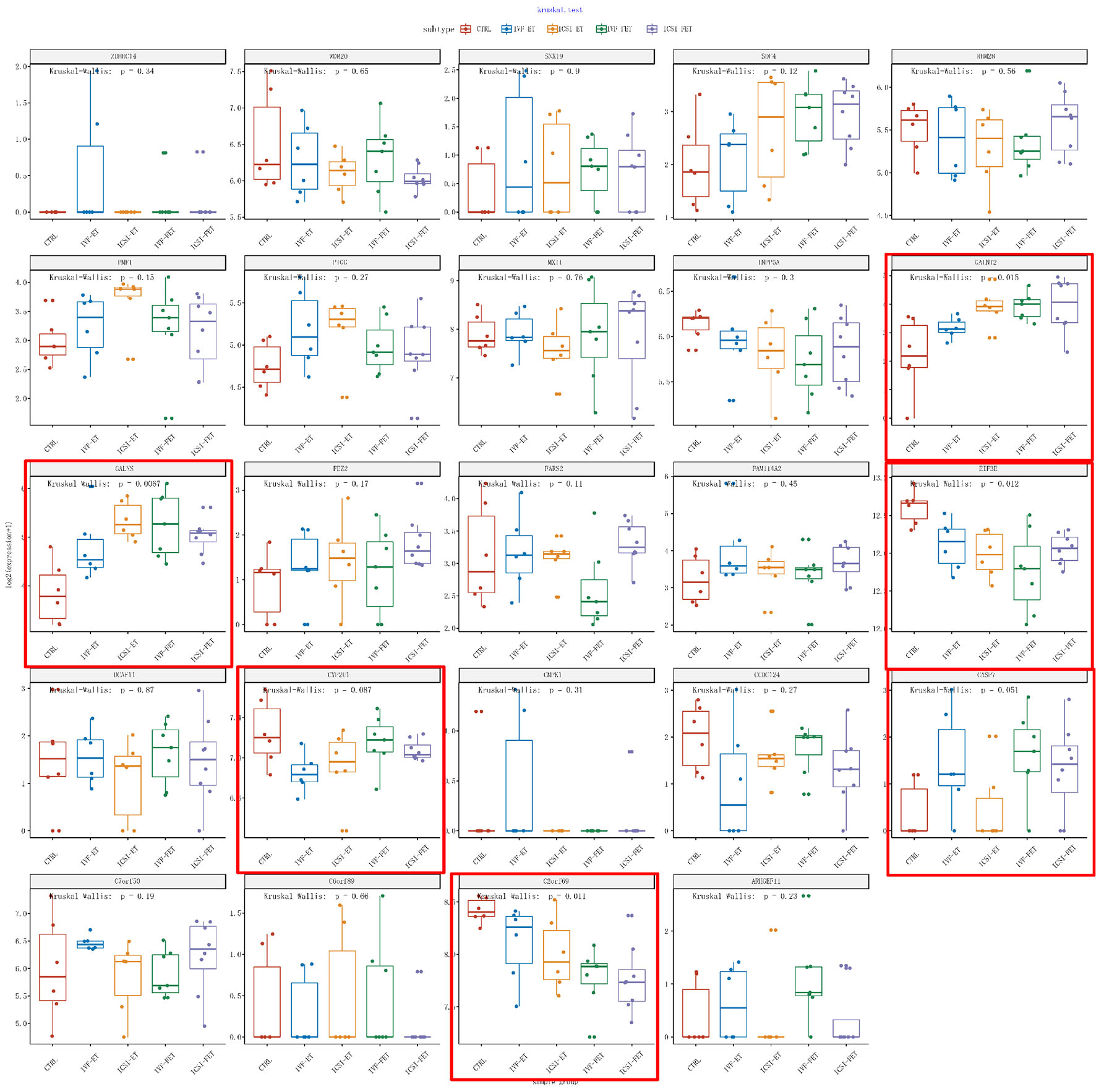

Supplement: Supplementary Figure 4 — House keeping genes and imprinting genes in DMSs was confirmed by RNA-seq. The expression profiles of GALNT2, GALNS, EIF3E, C2ORF69, CYP2U1, and CASP7 in the four ART subgroups: IVF-ET, IVF-FET, ICSI-ET, and ICSI-FET, these six genes had significant differences in all ART subgroups. [file Image_4.JPEG]

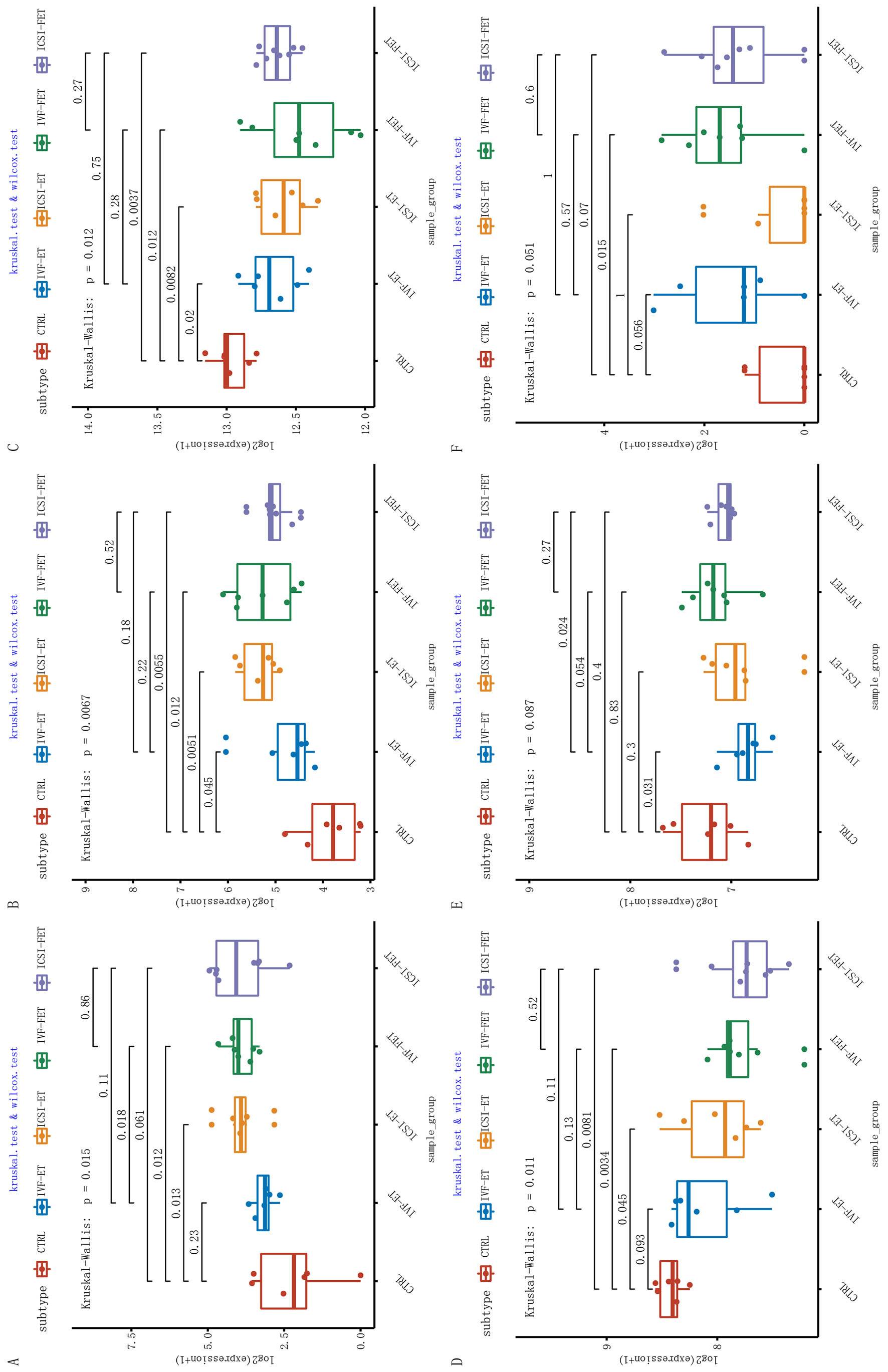

Supplement: Supplementary Figure 5 — Differentially expressed genes (DEGs) pathway analysis of different types of ART and natural pregnancy infants. DEGs pathway analysis showed the main different pathways, which were enriched in key pathways of immune-system and neuro-system, such as regulation of T cell activation, regulation of neural precursor cell proliferation and neuroblast proliferation et al. [file Image_5.JPEG]

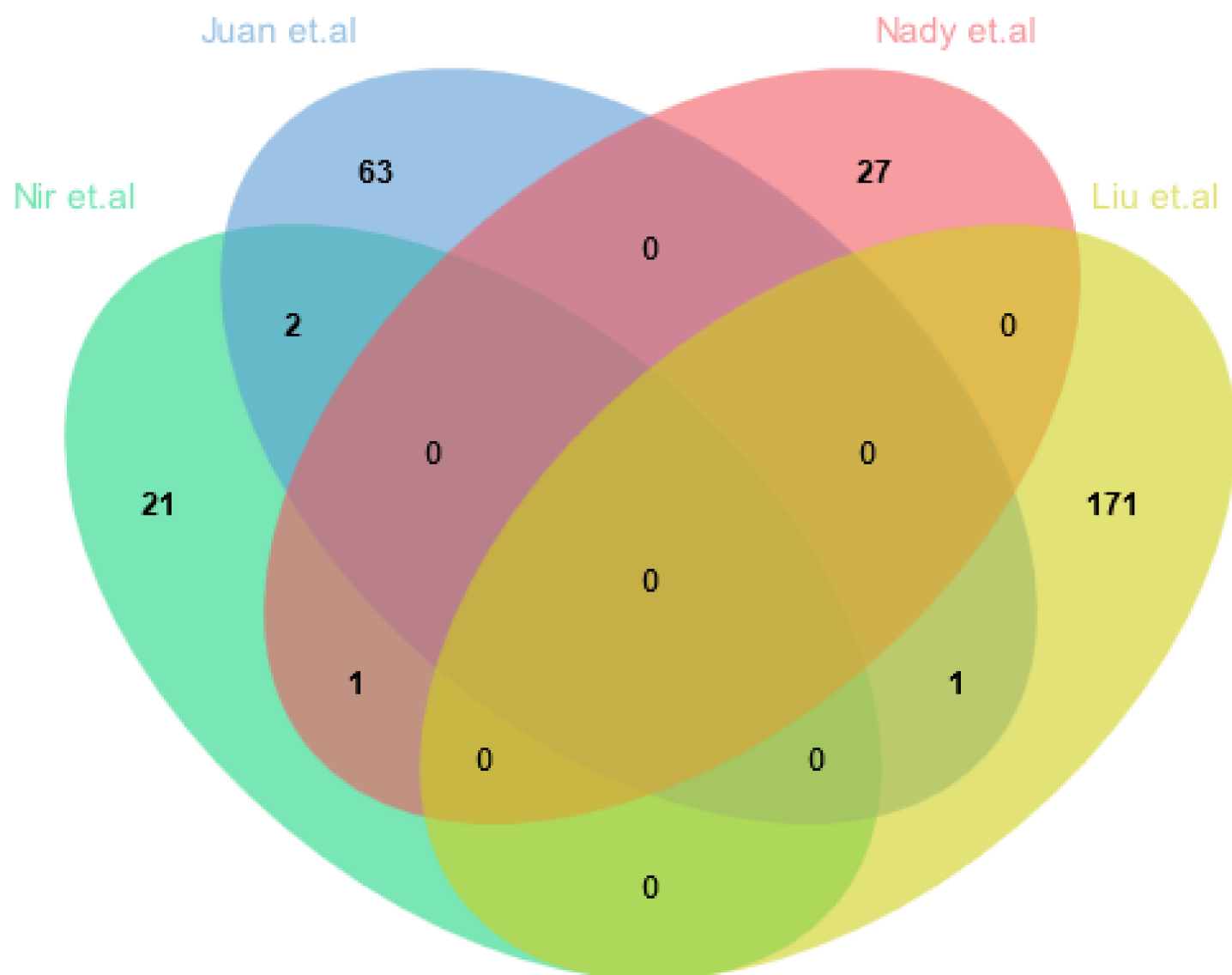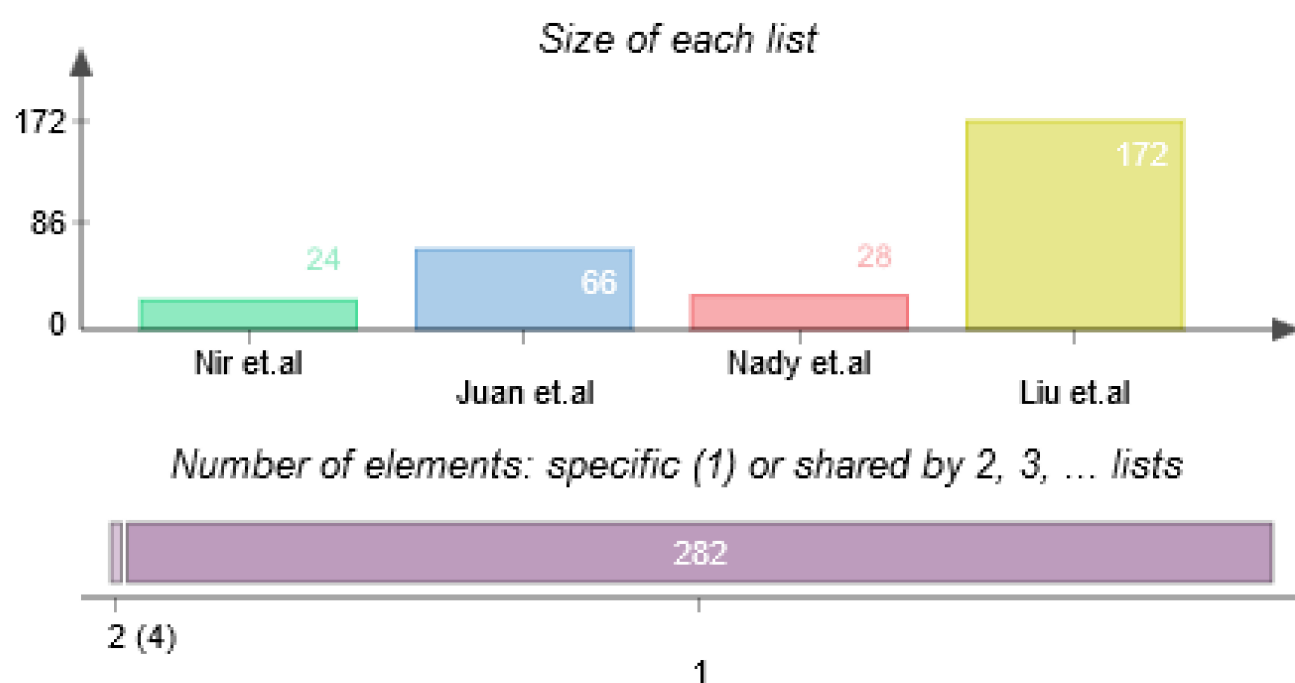

Supplement: Supplementary Figure 6 — Venn diagram showing the relationship with the reported genes. Venn diagram showed there were few overlaps in the differently methylated genes that have been reported. [file Data_Sheet_1.PDF]
