# Supplementary material for: Whole-Genome Methylation Analysis Revealed ART-Specific DNA Methylation Pattern of Neuro- and Immune-System Pathways in Chinese Human Neonates
Source: Front Genet. 2021 Sep 13;12:696840. doi: 10.3389/fgene.2021.696840 (PMC8473827; doi:10.3389/fgene.2021.696840)
Supplement: Supplementary file 7 [file Table_1.DOCX]

**Table S1 Statistical analysis of DMSs between two groups.**

| **cg ID** | **SD Control** | **Mean ART** | **SD ART** | **Heapmap Group** |
| --- | --- | --- | --- | --- |
| cg00729708 | 0.195485472 | 0.6739733 | 0.140937066 | C1 |
| cg00999163 | 0.011557594 | 0.483864933 | 0.237913871 | C1 |
| cg01726273 | 0.118407804 | 0.47370115 | 0.057229799 | C1 |
| cg02113055 | 0.220145538 | 0.757947 | 0.177456168 | C1 |
| cg02239258 | 0.041829483 | 0.507424933 | 0.104403381 | C1 |
| cg02730303 | 0.002588385 | 0.372782927 | 0.152752727 | C1 |
| cg03119308 | 0.006235517 | 0.7300495 | 0.176032644 | C1 |
| cg03979311 | 0.140942628 | 0.427873533 | 0.041040034 | C1 |
| cg04145539 | 0.044656885 | 0.532902467 | 0.015219886 | C1 |
| cg05376465 | 0.007812521 | 0.279100367 | 0.099942688 | C1 |
| cg06407043 | 0.034142711 | 0.426335153 | 0.261295851 | C1 |
| cg06915202 | 0.06615573 | 0.5307236 | 0.09192219 | C1 |
| cg06991380 | 0.179141786 | 0.487769933 | 0.038688032 | C1 |
| cg07093060 | 0.224215076 | 0.5735389 | 0.017197648 | C1 |
| cg08603678 | 0.010877162 | 0.739014933 | 0.185063182 | C1 |
| cg08684580 | 0.093725912 | 0.49294715 | 0.106792781 | C1 |
| cg08963013 | 0.00387379 | 0.533606917 | 0.014180916 | C1 |
| cg10590338 | 0.222842079 | 0.519998367 | 0.013847334 | C1 |
| cg10650398 | 0.145719775 | 0.4250483 | 0.015652674 | C1 |
| cg11418607 | 0.226312535 | 0.706084617 | 0.197003019 | C1 |
| cg12501287 | 0.174646605 | 0.43466645 | 0.145180004 | C1 |
| cg12604331 | 0.054556653 | 0.625649017 | 0.102360017 | C1 |
| cg12657416 | 0.015965819 | 0.5435133 | 0.28426921 | C1 |
| cg13294447 | 0.018069527 | 0.488393233 | 0.209017793 | C1 |
| cg14007688 | 0.205507646 | 0.646337328 | 0.296010698 | C1 |
| cg14065526 | 0.005628629 | 0.56736955 | 0.149342467 | C1 |
| cg14257199 | 0.033052075 | 0.43378935 | 0.148899124 | C1 |
| cg14444099 | 0.14022848 | 0.510939233 | 0.012479501 | C1 |
| cg16241932 | 0.206101193 | 0.521667562 | 0.280809824 | C1 |
| cg18576044 | 0.189935005 | 0.418629742 | 0.171856431 | C1 |
| cg18673341 | 0.222741697 | 0.550336905 | 0.386812188 | C1 |
| cg18838701 | 0.022551603 | 0.471934783 | 0.013735974 | C1 |
| cg19555075 | 0.196017047 | 0.632900233 | 0.168352054 | C1 |
| cg19577958 | 0.185720912 | 0.61162755 | 0.346336978 | C1 |
| cg20106077 | 0.054227762 | 0.380894483 | 0.311234138 | C1 |
| cg20823859 | 0.001528297 | 0.358794828 | 0.223034881 | C1 |
| cg20978247 | 0.17528986 | 0.5730812 | 0.108011678 | C1 |
| cg21519980 | 0.128520838 | 0.39066245 | 0.077484603 | C1 |
| cg22186694 | 0.1061943 | 0.462776117 | 0.022794829 | C1 |
| cg22996768 | 0.223565998 | 0.589690423 | 0.301315523 | C1 |
| cg23731272 | 0.101475231 | 0.55156855 | 0.152415448 | C1 |
| cg24307368 | 0.162612417 | 0.530942233 | 0.161117489 | C1 |
| cg25388952 | 0.092694807 | 0.602968983 | 0.108099837 | C1 |
| cg27597505 | 0.071851381 | 0.595172667 | 0.120457627 | C1 |
| cg00243527 | 0.307938243 | 0.69481449 | 0.290642796 | C2 |
| cg02882979 | 0.055723917 | 0.84392405 | 0.010735317 | C2 |
| cg03392100 | 0.016355084 | 0.865040683 | 0.017824629 | C2 |
| cg05464291 | 0.023027705 | 0.821893183 | 0.137663981 | C2 |
| cg05481961 | 0.007854887 | 0.889565867 | 0.009805075 | C2 |
| cg05554000 | 0.143052402 | 0.68459895 | 0.115885763 | C2 |
| cg05730108 | 0.204290545 | 0.583359733 | 0.136545723 | C2 |
| cg05900567 | 0.012746371 | 0.839094667 | 0.133907912 | C2 |
| cg06699489 | 0.289718203 | 0.82592375 | 0.024702375 | C2 |
| cg07134368 | 0.258806736 | 0.73798545 | 0.182310653 | C2 |
| cg08136432 | 0.341851527 | 0.781000233 | 0.259090538 | C2 |
| cg08198851 | 0.23560821 | 0.734299767 | 0.17860255 | C2 |
| cg08880261 | 0.010891223 | 0.78652895 | 0.156235343 | C2 |
| cg08912652 | 0.411689105 | 0.9302067 | 0.016931335 | C2 |
| cg08950364 | 0.015785634 | 0.738323917 | 0.131713387 | C2 |
| cg09339156 | 0.014727134 | 0.8874821 | 0.013032352 | C2 |
| cg13422830 | 0.021410519 | 0.934686983 | 0.013066027 | C2 |
| cg13444538 | 0.155859322 | 0.68724165 | 0.109375978 | C2 |
| cg13462557 | 0.2232664 | 0.761468533 | 0.182789789 | C2 |
| cg13641645 | 0.118415841 | 0.697838367 | 0.014244211 | C2 |
| cg14983172 | 0.287207683 | 0.697433633 | 0.028205641 | C2 |
| cg15567368 | 0.214337914 | 0.831840033 | 0.155717084 | C2 |
| cg16423096 | 0.049654457 | 0.727727783 | 0.171995424 | C2 |
| cg16987684 | 0.030288902 | 0.8267077 | 0.14482031 | C2 |
| cg19005275 | 0.018257576 | 0.848221833 | 0.014354924 | C2 |
| cg19650706 | 0.097931611 | 0.691372283 | 0.11087668 | C2 |
| cg20040891 | 0.010222315 | 0.769378483 | 0.154808996 | C2 |
| cg20979384 | 0.011065682 | 0.858703717 | 0.008553462 | C2 |
| cg23517115 | 0.313681245 | 0.8530068 | 0.012994966 | C2 |
| cg23517941 | 0.246894201 | 0.832825867 | 0.013995235 | C2 |
| cg23727079 | 0.010381851 | 0.841241383 | 0.005968474 | C2 |
| cg24158878 | 0.364262397 | 0.857192667 | 0.115248701 | C2 |
| cg24846009 | 0.015762722 | 0.729779217 | 0.171283581 | C2 |
| cg24976563 | 0.288637098 | 0.8124548 | 0.15093579 | C2 |
| cg25282454 | 0.009520812 | 0.717284867 | 0.01622375 | C2 |
| cg26069044 | 0.010446395 | 0.832363583 | 0.139392403 | C2 |
| cg26281303 | 0.40592588 | 0.9229099 | 0.013081751 | C2 |
| cg26889118 | 0.015656175 | 0.77285545 | 0.172486962 | C2 |
| cg00069771 | 0.176371813 | 0.9395064 | 0.011580259 | C3 |
| cg00084271 | 0.191064874 | 0.633431483 | 0.179850642 | C3 |
| cg00694040 | 0.180056331 | 0.731967917 | 0.140739882 | C3 |
| cg01138706 | 0.182645485 | 0.64431665 | 0.145988783 | C3 |
| cg01270299 | 0.337817146 | 0.939040483 | 0.020147673 | C3 |
| cg01551388 | 0.159350264 | 0.602072917 | 0.21219212 | C3 |
| cg01991743 | 0.198373759 | 0.89990225 | 0.012130691 | C3 |
| cg02379549 | 0.398259795 | 0.905307967 | 0.014111738 | C3 |
| cg03444934 | 0.357724284 | 0.85983755 | 0.028669114 | C3 |
| cg05385718 | 0.190998244 | 0.844676683 | 0.010324407 | C3 |
| cg05846337 | 0.143118576 | 0.8137166 | 0.02432356 | C3 |
| cg06314883 | 0.144610385 | 0.899137983 | 0.020851029 | C3 |
| cg06979386 | 0.209926717 | 0.620108917 | 0.167283654 | C3 |
| cg10625579 | 0.196427389 | 0.93136205 | 0.010000806 | C3 |
| cg10957001 | 0.184350933 | 0.917506867 | 0.015720506 | C3 |
| cg11074353 | 0.226669395 | 0.78739615 | 0.155391285 | C3 |
| cg11379315 | 0.319279449 | 0.867194383 | 0.018639263 | C3 |
| cg11420142 | 0.186657666 | 0.901843717 | 0.012605425 | C3 |
| cg11438287 | 0.168954311 | 0.834441267 | 0.012818346 | C3 |
| cg11474878 | 0.321585459 | 0.855803317 | 0.011661088 | C3 |
| cg14024893 | 0.081094208 | 0.789545917 | 0.077967887 | C3 |
| cg14068184 | 0.217711034 | 0.624574217 | 0.160903717 | C3 |
| cg14310831 | 0.157913966 | 0.829978817 | 0.020721233 | C3 |
| cg14609104 | 0.242040848 | 0.616988383 | 0.125674359 | C3 |
| cg14782559 | 0.310781309 | 0.775669617 | 0.139829041 | C3 |
| cg15532640 | 0.147638462 | 0.623060167 | 0.109014681 | C3 |
| cg18514595 | 0.223194939 | 0.7441375 | 0.180925256 | C3 |
| cg18863119 | 0.051786066 | 0.878326533 | 0.010089928 | C3 |
| cg19074779 | 0.165840679 | 0.934433583 | 0.014272144 | C3 |
| cg19393008 | 0.012272099 | 0.741107267 | 0.186526341 | C3 |
| cg20865618 | 0.392786412 | 0.923343467 | 0.054544848 | C3 |
| cg22136749 | 0.159546811 | 0.797496267 | 0.008392722 | C3 |
| cg22274273 | 0.206177205 | 0.6665547 | 0.183364826 | C3 |
| cg22408108 | 0.153210676 | 0.803325933 | 0.01275343 | C3 |
| cg22626897 | 0.328558271 | 0.8688939 | 0.017400485 | C3 |
| cg24470466 | 0.331390075 | 0.923053783 | 0.023265437 | C3 |
| cg24739935 | 0.045198671 | 0.851908333 | 0.015461675 | C3 |
| cg25543264 | 0.133043782 | 0.796792867 | 0.017937927 | C3 |
| cg26180843 | 0.138270064 | 0.825913033 | 0.033325717 | C3 |
| cg26843567 | 0.157660003 | 0.8956697 | 0.015120771 | C3 |
| cg27577781 | 0.260467088 | 0.695201083 | 0.214212691 | C3 |
| cg00320354 | 0.012317253 | 0.676682233 | 0.16817747 | C4 |
| cg00587941 | 0.008946907 | 0.538874687 | 0.364534587 | C4 |
| cg02288345 | 0.011446484 | 0.501120067 | 0.159019054 | C4 |
| cg02619116 | 0.004513152 | 0.630056717 | 0.295426927 | C4 |
| cg04132017 | 0.009210612 | 0.645727768 | 0.318227814 | C4 |
| cg04798314 | 0.021770176 | 0.69607025 | 0.158660007 | C4 |
| cg04835511 | 0.009752666 | 0.47210613 | 0.34834796 | C4 |
| cg05879576 | 0.014336636 | 0.574716633 | 0.309086436 | C4 |
| cg05893845 | 0.010443412 | 0.679501617 | 0.164368189 | C4 |
| cg05990366 | 0.007503708 | 0.653802017 | 0.20421776 | C4 |
| cg06844845 | 0.03822135 | 0.570176232 | 0.332763304 | C4 |
| cg06995503 | 0.008696792 | 0.59704805 | 0.187727305 | C4 |
| cg07270289 | 0.01273153 | 0.641175633 | 0.163534309 | C4 |
| cg07387335 | 0.009530842 | 0.6723591 | 0.131889242 | C4 |
| cg07523712 | 0.025257072 | 0.645331117 | 0.137383679 | C4 |
| cg08049519 | 0.005095338 | 0.648863267 | 0.171680696 | C4 |
| cg08594246 | 0.019846114 | 0.60628695 | 0.141373476 | C4 |
| cg09614301 | 0.01799783 | 0.5736585 | 0.182766577 | C4 |
| cg09627057 | 0.004901795 | 0.6492699 | 0.192151276 | C4 |
| cg09663736 | 0.009299563 | 0.6080374 | 0.142626604 | C4 |
| cg09727206 | 0.001960033 | 0.559432317 | 0.149508545 | C4 |
| cg09762182 | 0.008009337 | 0.710034467 | 0.161658948 | C4 |
| cg10246121 | 0.018449238 | 0.56822915 | 0.158832818 | C4 |
| cg10280308 | 0.008421537 | 0.721279883 | 0.303041663 | C4 |
| cg11539066 | 0.069586243 | 0.587556417 | 0.054849141 | C4 |
| cg11547201 | 0.003770161 | 0.596619267 | 0.12179877 | C4 |
| cg11663691 | 0.010613379 | 0.434908592 | 0.317649438 | C4 |
| cg11723923 | 0.014162444 | 0.59841495 | 0.186404746 | C4 |
| cg13033971 | 0.018017055 | 0.733324117 | 0.010578949 | C4 |
| cg14060113 | 0.107715875 | 0.545493033 | 0.309031845 | C4 |
| cg14464852 | 0.017886066 | 0.65901615 | 0.20678414 | C4 |
| cg14497649 | 0.002682867 | 0.622698267 | 0.006793434 | C4 |
| cg15270561 | 0.015217411 | 0.639478 | 0.357593934 | C4 |
| cg15677681 | 0.013173403 | 0.655770817 | 0.184536074 | C4 |
| cg16515381 | 0.007558626 | 0.61501104 | 0.368084848 | C4 |
| cg16542356 | 0.010031579 | 0.75330215 | 0.26775492 | C4 |
| cg16645815 | 0.015746497 | 0.653175383 | 0.166114203 | C4 |
| cg16738827 | 0.018056034 | 0.707480583 | 0.148027153 | C4 |
| cg17004290 | 0.014995599 | 0.483948062 | 0.352744757 | C4 |
| cg17406915 | 0.022827967 | 0.438273413 | 0.303525604 | C4 |
| cg17658874 | 0.034014937 | 0.4844934 | 0.252966447 | C4 |
| cg19156046 | 0.022934927 | 0.567273132 | 0.331287855 | C4 |
| cg19880901 | 0.006101012 | 0.562751683 | 0.194712731 | C4 |
| cg22237495 | 0.022584046 | 0.54988708 | 0.267475376 | C4 |
| cg22481673 | 0.013249103 | 0.649067967 | 0.191722697 | C4 |
| cg22645355 | 0.007022071 | 0.471321817 | 0.360906262 | C4 |
| cg23101028 | 0.069865568 | 0.63181735 | 0.024806927 | C4 |
| cg23168520 | 0.012204647 | 0.58905775 | 0.232088911 | C4 |
| cg23432430 | 0.008922526 | 0.644171583 | 0.193059982 | C4 |
| cg23495837 | 0.061019467 | 0.450647228 | 0.292141189 | C4 |
| cg23496178 | 0.020248521 | 0.532360837 | 0.344984394 | C4 |
| cg23499373 | 0.011495846 | 0.5673624 | 0.142866614 | C4 |
| cg24051749 | 0.01410431 | 0.512807817 | 0.156465591 | C4 |
| cg24412204 | 0.016902962 | 0.649425067 | 0.152683895 | C4 |
| cg25465065 | 0.018357706 | 0.529380912 | 0.374978623 | C4 |
| cg26796043 | 0.003817742 | 0.648488317 | 0.201531682 | C4 |
| cg27244972 | 0.090476443 | 0.536153583 | 0.214540612 | C4 |
| cg00023507 | 0.022209032 | 0.43957925 | 0.154196279 | C5 |
| cg00209612 | 0.166453788 | 0.454654533 | 0.005866359 | C5 |
| cg00540941 | 0.027658311 | 0.49300925 | 0.09537446 | C5 |
| cg00660167 | 0.118959502 | 0.503090133 | 0.086743241 | C5 |
| cg00681003 | 0.027854374 | 0.4988359 | 0.01452758 | C5 |
| cg00968488 | 0.153575621 | 0.279567498 | 0.316258821 | C5 |
| cg01347786 | 0.019214609 | 0.416869495 | 0.296186845 | C5 |
| cg02096172 | 0.122782451 | 0.5537615 | 0.056644977 | C5 |
| cg03965172 | 0.193662621 | 0.488414983 | 0.015574247 | C5 |
| cg04130408 | 0.016454431 | 0.346104562 | 0.138431892 | C5 |
| cg04331561 | 0.020371482 | 0.361524575 | 0.225723209 | C5 |
| cg04741728 | 0.026423708 | 0.563389983 | 0.185959346 | C5 |
| cg05401945 | 0.043218802 | 0.420703468 | 0.170350531 | C5 |
| cg05418105 | 0.120432329 | 0.5350107 | 0.075436371 | C5 |
| cg06675417 | 0.079605565 | 0.498588067 | 0.191091544 | C5 |
| cg07711085 | 0.114772935 | 0.596534833 | 0.174348619 | C5 |
| cg07903626 | 0.034994816 | 0.250144647 | 0.214549126 | C5 |
| cg08600378 | 0.022820959 | 0.475217133 | 0.141494326 | C5 |
| cg09856996 | 0.012469063 | 0.539245217 | 0.13158859 | C5 |
| cg10486069 | 0.058530992 | 0.336775783 | 0.077948383 | C5 |
| cg11577454 | 0.014618706 | 0.60979968 | 0.288943794 | C5 |
| cg11680857 | 0.026485283 | 0.529513015 | 0.25167803 | C5 |
| cg11857805 | 0.179066551 | 0.356720582 | 0.23411325 | C5 |
| cg12765123 | 0.106328334 | 0.5203539 | 0.075321897 | C5 |
| cg12823233 | 0.087445547 | 0.547931417 | 0.053127939 | C5 |
| cg13143872 | 0.03482061 | 0.458884033 | 0.031560302 | C5 |
| cg13211008 | 0.155452528 | 0.386485083 | 0.145811344 | C5 |
| cg15295200 | 0.176892725 | 0.197039745 | 0.213555207 | C5 |
| cg15460035 | 0.020915281 | 0.448549182 | 0.167297503 | C5 |
| cg16046605 | 0.023371578 | 0.400440493 | 0.210028481 | C5 |
| cg16106427 | 0.078623458 | 0.40498705 | 0.128253115 | C5 |
| cg16320888 | 0.025366267 | 0.40847995 | 0.206933921 | C5 |
| cg16435686 | 0.026582544 | 0.42819962 | 0.216183718 | C5 |
| cg16836675 | 0.175088069 | 0.386324827 | 0.236620595 | C5 |
| cg18105134 | 0.008506216 | 0.566003867 | 0.169759353 | C5 |
| cg19188207 | 0.041446794 | 0.519669033 | 0.16093185 | C5 |
| cg19375418 | 0.099178957 | 0.530469883 | 0.125578937 | C5 |
| cg19455396 | 0.009168348 | 0.251920767 | 0.29758885 | C5 |
| cg19754622 | 0.161028925 | 0.437146267 | 0.111116443 | C5 |
| cg20036791 | 0.019473401 | 0.437438167 | 0.116400235 | C5 |
| cg20336730 | 0.053044029 | 0.506153683 | 0.035210735 | C5 |
| cg20360416 | 0.145204776 | 0.507070283 | 0.023361185 | C5 |
| cg22901297 | 0.039660074 | 0.466438267 | 0.205097527 | C5 |
| cg22999620 | 0.07739382 | 0.465558367 | 0.299078807 | C5 |
| cg23162598 | 0.017104089 | 0.49624039 | 0.306919601 | C5 |
| cg23687434 | 0.006499406 | 0.446929857 | 0.18422484 | C5 |
| cg25450321 | 0.038140532 | 0.210624963 | 0.253430687 | C5 |
| cg26056277 | 0.093118862 | 0.5457878 | 0.124430621 | C5 |
| cg26512469 | 0.168532476 | 0.37459976 | 0.224232809 | C5 |
| cg26853071 | 0.006528698 | 0.4603017 | 0.119181435 | C5 |
| cg27014438 | 0.016603651 | 0.513539423 | 0.258701939 | C5 |
| cg27639199 | 0.004972107 | 0.370917495 | 0.218134351 | C5 |
| cg00597445 | 0.02030084 | 0.343006783 | 0.233119611 | C6 |
| cg00740510 | 0.328169055 | 0.189271538 | 0.238111947 | C6 |
| cg01128042 | 0.171569462 | 0.17203075 | 0.322179034 | C6 |
| cg01350803 | 0.168266231 | 0.110538327 | 0.188018371 | C6 |
| cg02157463 | 0.01848142 | 0.19662948 | 0.150429264 | C6 |
| cg02188142 | 0.036298399 | 0.097586858 | 0.02252749 | C6 |
| cg02314201 | 0.108402943 | 0.22860925 | 0.071908328 | C6 |
| cg02487331 | 0.092228773 | 0.3130999 | 0.117617606 | C6 |
| cg02952913 | 0.065299095 | 0.320218183 | 0.087231527 | C6 |
| cg02964324 | 0.013381324 | 0.286583317 | 0.116769322 | C6 |
| cg03526459 | 0.115113145 | 0.274908817 | 0.13006182 | C6 |
| cg03938978 | 0.011953485 | 0.1377633 | 0.033058914 | C6 |
| cg04089743 | 0.119362556 | 0.325765368 | 0.112570633 | C6 |
| cg04572930 | 0.104694223 | 0.327778767 | 0.100581976 | C6 |
| cg05057827 | 0.15023517 | 0.245454917 | 0.100027147 | C6 |
| cg05398700 | 0.107533857 | 0.426191983 | 0.108413757 | C6 |
| cg05786381 | 0.274211401 | 0.2425326 | 0.071820675 | C6 |
| cg05890457 | 0.202274114 | 0.21146273 | 0.195407623 | C6 |
| cg05971102 | 0.249148451 | 0.0413326 | 0.024805844 | C6 |
| cg06634576 | 0.201137005 | 0.25489661 | 0.164069537 | C6 |
| cg06758191 | 0.146781006 | 0.205004618 | 0.260855555 | C6 |
| cg07028768 | 0.197155714 | 0.184735308 | 0.1951003 | C6 |
| cg07350262 | 0.165016734 | 0.2520008 | 0.074087312 | C6 |
| cg07600533 | 0.047313149 | 0.1253765 | 0.013831176 | C6 |
| cg07791065 | 0.165510271 | 0.421154513 | 0.167500081 | C6 |
| cg07878625 | 0.007661045 | 0.342657483 | 0.368759204 | C6 |
| cg08425796 | 0.118287312 | 0.401241033 | 0.089490939 | C6 |
| cg08506353 | 0.136146621 | 0.2306764 | 0.095929506 | C6 |
| cg09329516 | 0.167665476 | 0.131000002 | 0.028722211 | C6 |
| cg09417038 | 0.101336725 | 0.431758617 | 0.100513798 | C6 |
| cg10071929 | 0.103546603 | 0.154025995 | 0.092572994 | C6 |
| cg10690440 | 0.096381767 | 0.3751513 | 0.043682798 | C6 |
| cg11395954 | 0.065887729 | 0.344738667 | 0.050843329 | C6 |
| cg11725415 | 0.03572688 | 0.3744532 | 0.176141409 | C6 |
| cg11960243 | 0.010520101 | 0.40300365 | 0.253829362 | C6 |
| cg12195446 | 0.192337985 | 0.315150997 | 0.204422151 | C6 |
| cg12556569 | 0.332743521 | 0.053492245 | 0.006763555 | C6 |
| cg13077366 | 0.098701643 | 0.35648065 | 0.063537745 | C6 |
| cg13150801 | 0.097192115 | 0.379772183 | 0.053667018 | C6 |
| cg13392078 | 0.105524784 | 0.186857733 | 0.027347381 | C6 |
| cg13748354 | 0.153640806 | 0.10496164 | 0.178173802 | C6 |
| cg14390143 | 0.149348082 | 0.32338285 | 0.119468543 | C6 |
| cg15019001 | 0.140145097 | 0.3701867 | 0.097201202 | C6 |
| cg15122621 | 0.12832196 | 0.363278 | 0.137245248 | C6 |
| cg15421137 | 0.368366969 | 0.053635752 | 0.008343985 | C6 |
| cg16335098 | 0.209841828 | 0.273706617 | 0.077220586 | C6 |
| cg16814483 | 0.080956706 | 0.205609352 | 0.101984898 | C6 |
| cg16871435 | 0.025294594 | 0.10676764 | 0.135143346 | C6 |
| cg17738613 | 0.008963693 | 0.140153023 | 0.175191228 | C6 |
| cg19214707 | 0.010311698 | 0.183471653 | 0.190356256 | C6 |
| cg19389973 | 0.182344085 | 0.262645682 | 0.223177829 | C6 |
| cg20094343 | 0.064567118 | 0.099949185 | 0.10616836 | C6 |
| cg20356878 | 0.034170279 | 0.471360883 | 0.17535735 | C6 |
| cg20592836 | 0.172933186 | 0.06015811 | 0.007087908 | C6 |
| cg21263664 | 0.073927579 | 0.319696467 | 0.134538479 | C6 |
| cg22249529 | 0.050879489 | 0.160225833 | 0.039020449 | C6 |
| cg22862357 | 0.256011659 | 0.161654882 | 0.194445761 | C6 |
| cg23128510 | 0.027644912 | 0.040756103 | 0.007764492 | C6 |
| cg23513018 | 0.025037428 | 0.136820173 | 0.203615864 | C6 |
| cg23907051 | 0.078958643 | 0.332620217 | 0.09818312 | C6 |
| cg24080129 | 0.018305954 | 0.121693918 | 0.161821103 | C6 |
| cg24833027 | 0.179773272 | 0.349352433 | 0.140054316 | C6 |
| cg24849373 | 0.178760134 | 0.412119517 | 0.162557102 | C6 |
| cg24853868 | 0.074239396 | 0.2954096 | 0.099199165 | C6 |
| cg25977769 | 0.309207269 | 0.09596779 | 0.145723549 | C6 |
| cg26303777 | 0.01737137 | 0.263553795 | 0.223845842 | C6 |
| cg27084952 | 0.045685367 | 0.36564995 | 0.103110904 | C6 |
| cg27286614 | 0.306577139 | 0.243955067 | 0.175028595 | C6 |
| cg27321466 | 0.025350622 | 0.201802057 | 0.113529113 | C6 |
