# Supplementary material for: Whole-Genome Methylation Analysis Revealed ART-Specific DNA Methylation Pattern of Neuro- and Immune-System Pathways in Chinese Human Neonates
Source: Front Genet. 2021 Sep 13;12:696840. doi: 10.3389/fgene.2021.696840 (PMC8473827; doi:10.3389/fgene.2021.696840)
Supplement: Supplementary file 8 [file Table_2.DOCX]

**Supplementary Table 2 The detailed information of the most vulnerable methylation sites in IVF**

| **TargetID** | **CHR** | **MAPINFO** | **Gene Name** | **Diff（IVF-N）** | **P-Value** |
| --- | --- | --- | --- | --- | --- |
| cg01270299 | 19 | 53099757 | *ZNF137* | 0.58 | 0.002 |
| cg19455396 | 6 | 32796056 | *TAP2* | -0.67 | 0.002 |
| cg03119308 | 7 | 127950724 | *RBM28* | 0.68 | 0.002 |
| cg13748354 | 7 | 2289888 | *NUDT1* | -0.69 | 0.002 |
| cg15295200 | 3 | 139397901 | *NMNAT3* | -0.56 | 0.004 |
| cg08603678 | 8 | 109235928 | *EIF3E* | 0.69 | 0.002 |
| cg06758191 | 4 | 7812988 | *AFAP1* | -0.61 | 0.002 |
| cg23128510 | 2 | 175922785 | *-* | -0.86 | 0.002 |
| cg15567368 | 7 | 563891 | *-* | 0.65 | 0.002 |
| cg07903626 | 16 | 66098650 | *-* | -0.57 | 0.002 |
